# Selection of a novel strain of *Christensenella minuta* as a future biotherapy for Crohn’s disease

**DOI:** 10.1038/s41598-022-10015-3

**Published:** 2022-04-11

**Authors:** Karima Relizani, Katy Le Corf, Camille Kropp, Rebeca Martin-Rosique, Déborah Kissi, Guillaume Déjean, Lisa Bruno, Ccori Martinez, Georges Rawadi, Frédéric Elustondo, Wilfrid Mazier, Sandrine P. Claus

**Affiliations:** 1 Ysopia Bioscience, 17 place de la Bourse, 33076 Bordeaux, France; 2 Micalis Institute, AgroParisTech, INRAE, Université Paris-Saclay, 78350 Jouy-en-Josas, France

**Keywords:** Microbiome, Drug development

## Abstract

Microbiome-based therapies for inflammatory bowel diseases offer a novel and promising therapeutic approach. The human commensal bacteria of the species *Christensenella minuta* (*C. minuta*) have been reported consistently missing in patients affected by Crohn’s disease (CD) and have been documented to induce anti-inflammatory effects in human epithelial cells, supporting their potential as a novel biotherapy. This work aimed at selecting the most promising strain of *C. minuta* for future development as a clinical candidate for CD therapy. Here, we describe a complete screening process combining in vitro and in vivo assays to conduct a rational selection of a live strain of *C. minuta* with strong immunomodulatory properties. Starting from a collection of 32 strains, a panel of in vitro screening assays was used to narrow it down to five preclinical candidates that were further screened in vivo in an acute TNBS-induced rat colitis model. The most promising candidate was validated in vivo in two mouse models of colitis. The validated clinical candidate strain, *C. minuta* DSM 33715, was then fully characterized. Hence, applying a rationally designed screening algorithm, a novel strain of *C. minuta* was successfully identified as the most promising clinical candidate for CD.

## Introduction

Inflammatory Bowel Diseases (IBD) are chronic inflammatory conditions affecting the entire gastrointestinal tract and characterized by an immune overactivity. The main forms of the condition are known as ulcerative colitis (UC) and Crohn’s disease (CD), the latter being a debilitating and incurable disease characterized by patchy inflammation of the gut mucosa^1^.

The causes of the disease are not well understood but it is admitted that there is a gut microbiota dysbiosis in patients with active disease. Moreover, it has been demonstrated that the dysbiosis of treatment-naïve CD patients is similar to those observed in adults with long-standing treatment, indicating that the gut dysbiosis of CD patients is not caused by treatments and may precede disease development^2^. Another feature of CD-associated gut microbiota is its instability in the quiescent phase of the disease, just before relapse^3^. Accordingly, gut microbiome-derived therapies are increasingly gaining attention to stabilize the microbial ecosystem and prolong remission periods^4^.

The *Christensenellaceae* is a family of bacteria that was first described in 2012^5^ and the first genome of the type strain *Christensenella minuta* (*C. minuta*) DSM 22607 was published in 2017^6^. The same year, the *Christensenellaceae* were reported among the main predictive taxa significantly decreased in a cohort of CD patients^7^. Since then, a systematic loss of *Christensenellaceae* in CD patients has been reported in several studies^3,8–11^. In addition, Braun et al. demonstrated that the gut microbial instability before CD flare is characterized by a sudden loss of *Christensenellaceae* preceding disease recurrence^3^. Remarkably, the authors used the relative abundance of *Christensenellaceae*, along decrease in S24.7 bacteria and increase in *Gemellaceae,* to make a predictive index of CD flare that performed better than any other clinical marker currently used in clinics. In addition, it has been reported that *Christensenellaceae* were increased in people expressing the protective variant of the IL23R gene^10^, a well-known risk factor of CD, suggesting that these taxa may play an important role in disease prevention. Together, these indicate a role for *Christensenellaceae* loss as one of the pathogenic factors contributing to CD etiology. Based on this rationale, we previously published a first proof-of-concept work where we demonstrated the anti-inflammatory potential of the type strain *C. minuta* DSM 22607 in rodent colitis models^12^.

This follow-up work describes the screening process applied to select a potent *C. minuta* strain from an internal private culture collection to further develop it as a live biotherapeutic product to relieve CD-associated symptoms and slow down disease progression. The strain was further described and registered as *C. minuta* DSM 33715.

## Results

### Description of the *C. minuta* collection and screening strategy

**Figure 1:**
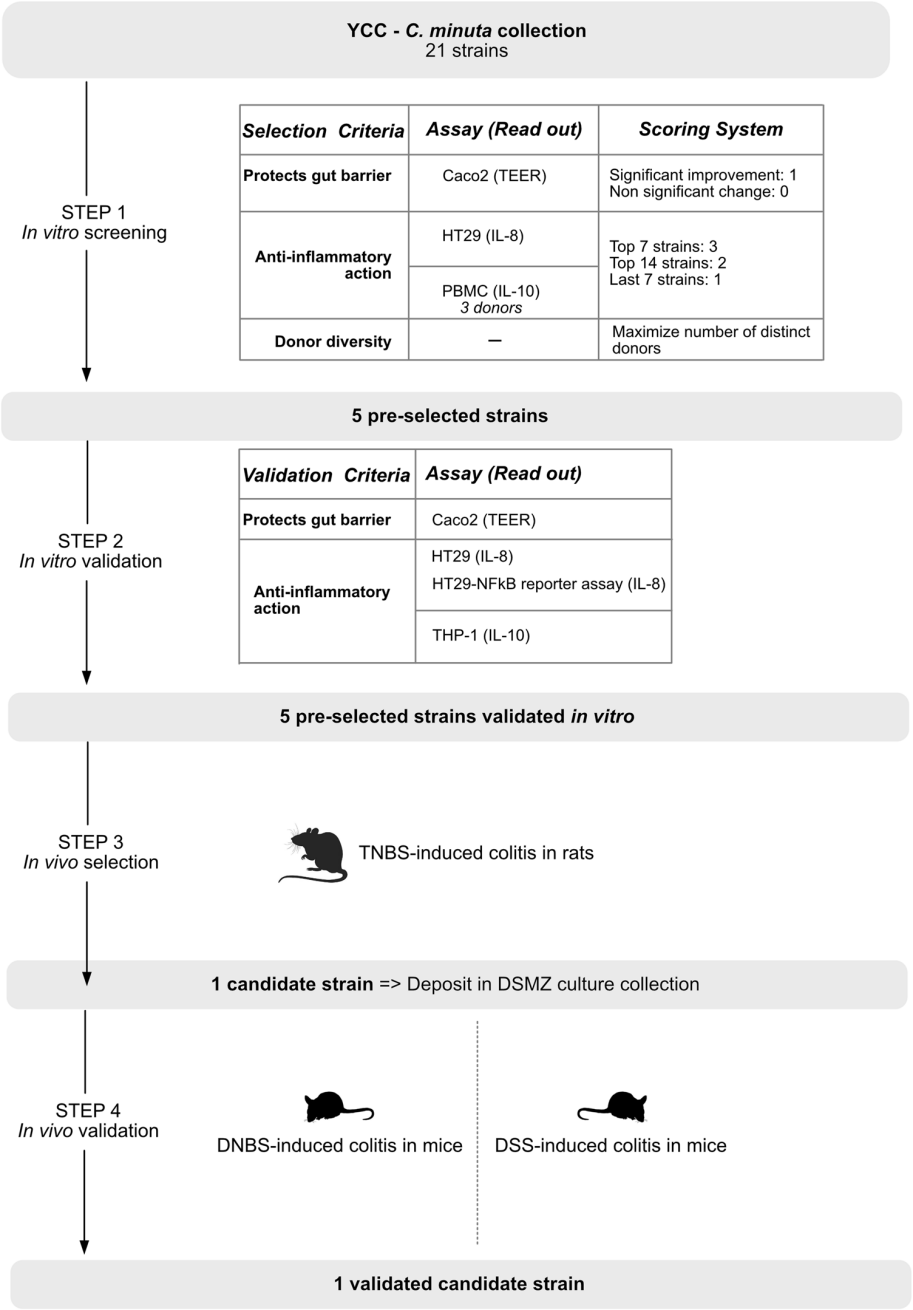
Schematic representation of the screening process.

The collection of *C. minuta* strains used for this study contained 32 strains all derived from healthy human donors. The screening strategy aimed at identifying a lead drug candidate addressing two prominent features of CD: an impaired gut inflammation and an impaired intestinal barrier integrity. The four-step screening process is described in Fig. 1. The first step included four in vitro assays (STEP 1) aiming to narrow down the number of strains to 5 pre-selected strains. The screening criteria were the following: (1) ability to grow in the culture medium selected for screening; (2) protective action on intestinal permeability; (3) beneficial immunomodulatory potential; (4) maximization of donor diversity to avoid clones. For all in vitro tests, the type-strain *C. minuta* DSM 22607 was used as a control reference. As illustrated in Fig. 1, we established a scoring system to identify the most promising strains. For the TEER assay assessing gut barrier integrity, a score of 1 was attributed to every strain that showed a significant improvement over the non-treated control and 0 otherwise. For scoring the anti-inflammatory response, we attributed a score of 3 for the top tier strains presenting the highest inhibitory effect on IL-8 production, a score of 2 to the strains in the second tier and a score of 1 to the lower tier. The same scoring was applied to IL-10 production.

Then the anti-inflammatory potential of the 5 STEP1-selected strains was evaluated in a follow-up series of in vitro assays (STEP 2). The 5 validated pre-selected strains were then assessed for efficacy in a validated in vivo model of colitis in rats^13^ (STEP 3). This step resulted in the selection of a strong clinical candidate strain with the highest efficacy in vivo. The in vivo efficacy of the selected clinical candidate strain was finally confirmed in distinct in vivo models of colitis in mice (STEP 4).

**STEP 1: in vitro screening of the** ***C. minuta*** **strain collection**

This step aimed at pre-selecting 5 lead drug candidates out of the original collection of 32 strains. Three strains had already been selected for other development programs and were not included in the initial screening.

First, we screened the remaining 29 strains based on their ability to grow in the culture medium selected for further assays (GAMm). Eight strains were thus eliminated based on slow growth (Table 1).

**Table 1:** Summary of the initial *C. minuta* collection and screening results.

*C. min *number	Donor	Growth	TEER	IL8	IL10 Donor a	IL10 Donor b	IL10 Donor g	Total score	Rank	Comment
**01**	**A**	**+++**	**1**	**2**	**2**	**3**	**2**	**10**	**5**	
02	B	+++	1	1	2	2	1	7	16	
03	C	+++	1	1	2	2	1	7	16	Donor already used in another programme
04	D	+++	1	1	1	3	1	7	16	Donor already used in another programme
**05**	**E**	**+++**	**1**	**3**	**2**	**2**	**2**	**10**	**5**	
06	F	+++	1	2	1	2	1	7	16	Donor already used in another programme
28	F	+/−	${{\slash\!\!\!\rm{O}}}$	${{\slash\!\!\!\rm{O}}}$	${{\slash\!\!\!\rm{O}}}$	${{\slash\!\!\!\rm{O}}}$	${{\slash\!\!\!\rm{O}}}$	${{\slash\!\!\!\rm{O}}}$	${{\slash\!\!\!\rm{O}}}$	Slow growth
07	G	+++	1	3	3	2	1	10	5	
08	G	+++	1	2	1	1	1	6	21	
12	G	+	1	3	3	2	3	12	1	Moderate growth
14	G	+++	1	1	3	3	2	10	5	
15	G	+++	1	2	2	1	2	8	14	
16	G	+++	1	1	3	3	1	9	10	
17	G	+	1	1	3	3	3	11	3	Moderate growth
18	G	+++	1	2	1	2	2	8	14	
19	G	+++	1	2	1	1	2	7	16	
21	G	+++	1	3	2	1	3	10	5	
**22**	**G**	**+++**	**1**	**1**	**3**	**3**	**3**	**11**	**3**	
30	G	+/−	${{\slash\!\!\!\rm{O}}}$	${{\slash\!\!\!\rm{O}}}$	${{\slash\!\!\!\rm{O}}}$	${{\slash\!\!\!\rm{O}}}$	${{\slash\!\!\!\rm{O}}}$	${{\slash\!\!\!\rm{O}}}$	${{\slash\!\!\!\rm{O}}}$	Slow growth
**09**	**H**	**+++**	**1**	**3**	**3**	**3**	**2**	**12**	**1**	
20	H	+++	1	2	2	1	3	9	10	
29	H	+/−	${{\slash\!\!\!\rm{O}}}$	${{\slash\!\!\!\rm{O}}}$	${{\slash\!\!\!\rm{O}}}$	${{\slash\!\!\!\rm{O}}}$	${{\slash\!\!\!\rm{O}}}$	${{\slash\!\!\!\rm{O}}}$	${{\slash\!\!\!\rm{O}}}$	Slow growth
31	H	+/−	${{\slash\!\!\!\rm{O}}}$	${{\slash\!\!\!\rm{O}}}$	${{\slash\!\!\!\rm{O}}}$	${{\slash\!\!\!\rm{O}}}$	${{\slash\!\!\!\rm{O}}}$	${{\slash\!\!\!\rm{O}}}$	${{\slash\!\!\!\rm{O}}}$	Slow growth
10	I	+++	1	3	1	1	3	9	10	
11	I	+/−	${{\slash\!\!\!\rm{O}}}$	${{\slash\!\!\!\rm{O}}}$	${{\slash\!\!\!\rm{O}}}$	${{\slash\!\!\!\rm{O}}}$	${{\slash\!\!\!\rm{O}}}$	${{\slash\!\!\!\rm{O}}}$	${{\slash\!\!\!\rm{O}}}$	Slow growth
**13**	**I**	**+++**	**1**	**3**	**1**	**1**	**3**	**9**	**10**	
23	I	+/−	${{\slash\!\!\!\rm{O}}}$	${{\slash\!\!\!\rm{O}}}$	${{\slash\!\!\!\rm{O}}}$	${{\slash\!\!\!\rm{O}}}$	${{\slash\!\!\!\rm{O}}}$	${{\slash\!\!\!\rm{O}}}$	${{\slash\!\!\!\rm{O}}}$	Slow growth
24	I	+/−	${{\slash\!\!\!\rm{O}}}$	${{\slash\!\!\!\rm{O}}}$	${{\slash\!\!\!\rm{O}}}$	${{\slash\!\!\!\rm{O}}}$	${{\slash\!\!\!\rm{O}}}$	${{\slash\!\!\!\rm{O}}}$	${{\slash\!\!\!\rm{O}}}$	Slow growth
25	I	+/−	${{\slash\!\!\!\rm{O}}}$	${{\slash\!\!\!\rm{O}}}$	${{\slash\!\!\!\rm{O}}}$	${{\slash\!\!\!\rm{O}}}$	${{\slash\!\!\!\rm{O}}}$	${{\slash\!\!\!\rm{O}}}$	${{\slash\!\!\!\rm{O}}}$	Slow growth

Pre-selected strains after STEP 1 are shown in bold. ${{\slash\!\!\!\rm{O}}}$ indicates that the strain was not included in the screening.

**Figure 2:**
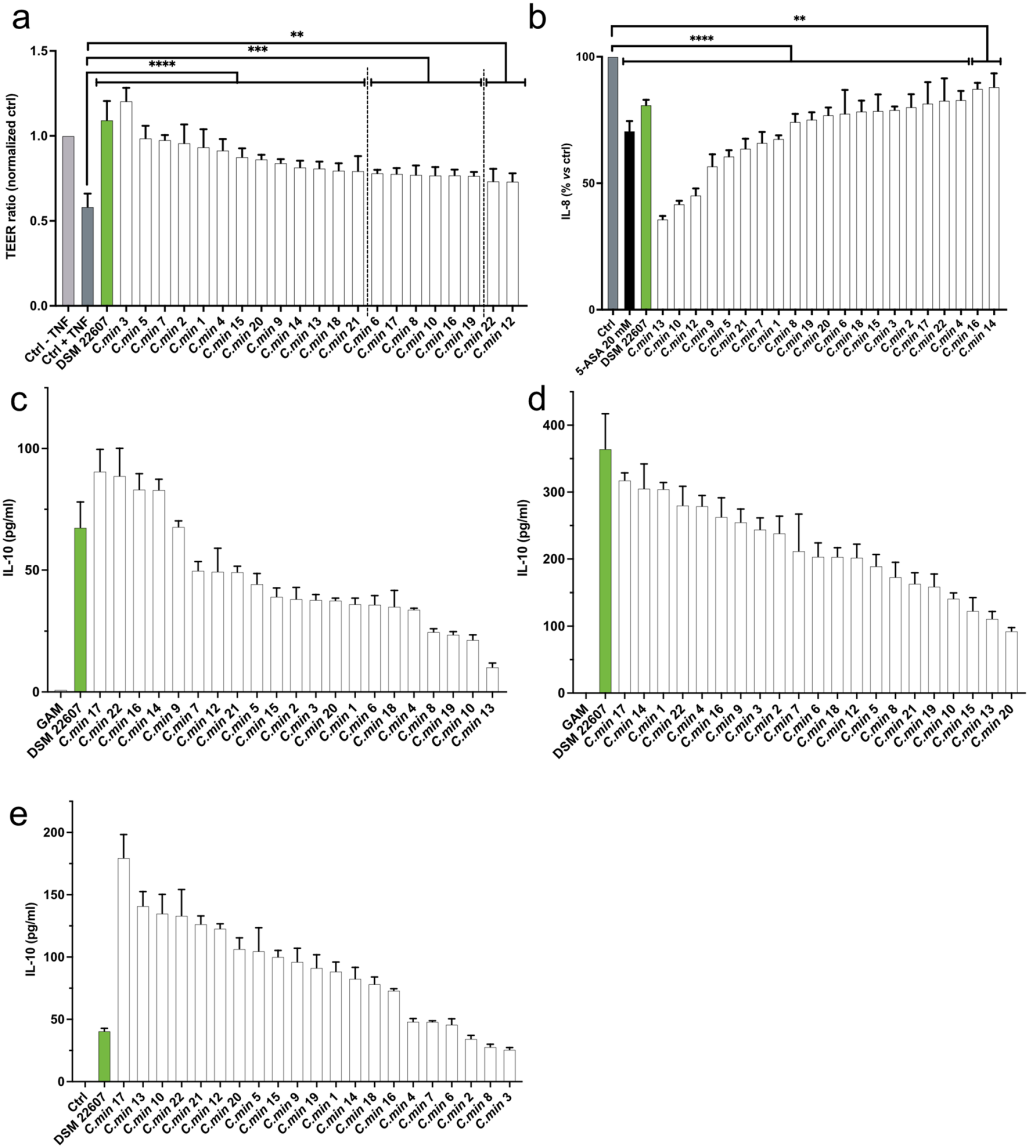
STEP 1 in vitro screening results: (**a**) Evaluation of the effects of our strains on intestinal barrier by measuring transepithelial electrical resistance (TEER). The polarized Caco-2 monolayers were challenged with TNF-α to disrupt the intestinal barrier. *Statistics*: one-way ANOVA followed by Dunnett’s multiple comparisons test (****p < 0.0001; ***p = 0.0005 for *C. min* 6, *p* = 0.0008 for *C. min* 8, *p* = 0.0003 for *C. min* 10 and 16, *p* = 0.0001 for *C. min* 17 and *p* = 0.0004 for *C. min* 19; **p = 0.0073 for *C. min* 12 and *p* = 0.0063 for *C. min* 22). (**b**) Evaluation of the anti-inflammatory properties on a human-derived intestinal cell line (HT-29) stimulated with TNF-α. *Statistics*: one-way ANOVA followed by Dunnett’s multiple comparison test: ****p < 0.0001, **p = 0.0029 for *C. min* 16 and p = 0.0059 *C. min* 14. 5-ASA 20 mM was used as a positive control. (**c**–**e**) IL-10 production by human-derived PBMCs on three different donors. No statistics were performed for this assay since we considered that any production of IL-10 was a positive response. *Key*: *Ctrl* Control with culture medium, *DSM 22607* *C. minuta* DSM 22607.

Second, to identify the strains with the highest potential to positively influence the immune response of the gut epithelium, we evaluated the protective effects of the remaining 21 strains on gut barrier permeability using TEER measured across a monolayer of human intestinal Caco-2 cells following a challenge with the pro-inflammatory cytokine TNF-α. All tested *C. minuta* strains had a powerful protective action on intestinal barrier integrity ranging from 73% (C min 12) to 120% (C min 3) of TEER (average 85% ± 1) compared to control (Fig. 2a). Third, the anti-inflammatory potential of the strains was evaluated through their ability to inhibit the production of the pro-inflammatory cytokine IL-8 by human intestinal cells following TNF-α stimulation. As illustrated on Fig. 2b, all strains displayed an anti-inflammatory action since they all limited IL-8 production (average inhibitory effect: 31.2 ± 14.6%). In particular, three strains (C min 13, 10 and 12) had a highly potent anti-inflammatory effect in this assay (64.25%, 58.24% and 54.79%, respectively). The systemic anti-inflammatory potential of the strains was also assessed through their ability to stimulate production of the anti-inflammatory cytokine IL-10 by PBMC cells in three donors. All strains induced a production of the anti-inflammatory cytokine IL-10 (Fig. 2c–e) and were therefore all considered positive for stimulation of IL-10.

Fourth, strains were organized by donor origin in order to avoid similar clones (Table 1). Finally, strains were scored as described and ranked accordingly (Table 1). Out of a maximum of 13 points, all tested strains scored between 7 and 12 points. *C. min* 9 (donor H) and *C. min* 12 (donor G) were the highest ranked strains (12 points) followed by *C. min* 17 and *C. min *22 (11 points), both from donor G. Since *C. min* 12 and *C. min* 17 showed a slightly less active growth than the other strains from donor G, we selected *C. min* 22 from this donor. *C. min* 9 was also selected from donor H. The next highest ranked strains in other donors were *C. min* 1 and *C. min* 5 from donors A and E, respectively. These were therefore also selected. Finally, the next high ranked strains were *C. min* 10 and *C. min* 13, both from donor I, which could not be discriminated from the preselection process. We chose to pursue with *C. min* 13. Hence, the five pre-selected strains at the end of STEP 1 were *C. min* 1, 5, 9, 13 and 22.

**STEP 2: in vitro confirmation of the five pre-selected candidate strains**

**Figure 3:**
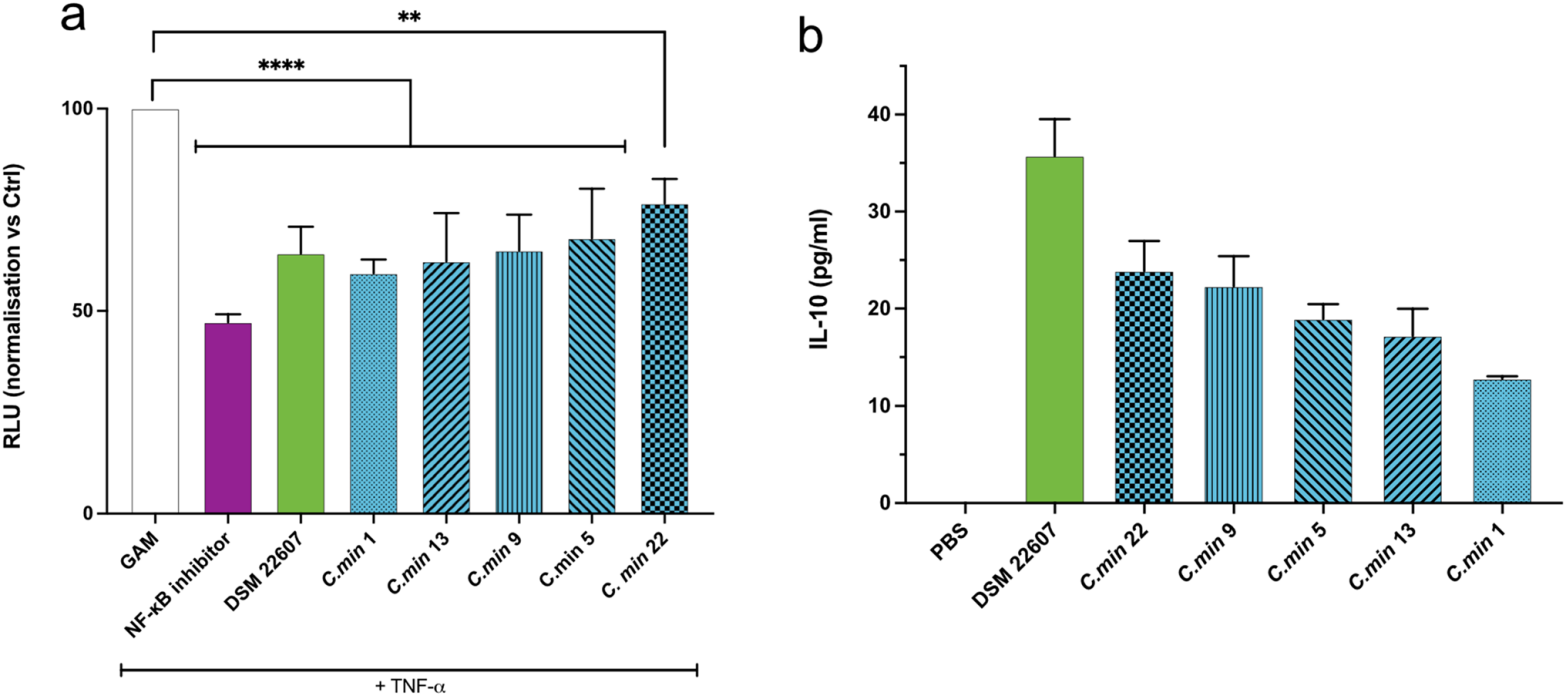
STEP 2 in vitro screening results confirmed the immunomodulatory action of the five pre-selected candidate strains. (**a**) Levels of NF-κB activation in HT-29 cells transfected with a reporter system and exposed to TNF-α. *Statistics*: one-way ANOVA followed by Dunnett’s multiple comparisons test ****p < 0.0001, **p = 0.0027 (**b**) IL-10 production by THP-1 cells differentiated in M0 macrophages by PMA treatment (100 ng/ml) in presence of *C. minuta* bacteria at MOI 50. No statistics were performed for this assay since any production of IL-10 was considered as a positive response.

The next step consisted in validating the anti-inflammatory action and protective effect on gut barrier integrity of the 5 pre-selected candidate strains. For this purpose, a new batch of all five strains was prepared to run the same assays as in STEP1. As expected, we were able to reproduce similar results for all pre-selected strains for inhibition of IL-8 production (Supplementary Fig. S1) and gut barrier protection (Supplementary Fig. S1). In addition, we performed two new in vitro assays. First, we tested the ability of our candidates to interact with the NF-κB pathway, which is known to play a key role in inflammation through the regulation of IL-8 production. As illustrated in Fig. 3A, all our candidates decreased the NF-κB activation induced by TNF-α stimulation (34.16 ± 5.9%), similarly to BAY11-7082, a NF-κB inhibitor. Second, we confirmed the ability of our candidates to induce IL-10 production by immune cells in PMA-differentiated THP1 cells (Fig. 3B).

**STEP 3: in vivo selection of the lead clinical candidate in a TNBS-induced colitis rat model**

We then pursued the screening process by investigating the efficacy of the five pre-selected strains in vivo in a TNBS-induced colitis model in rats, which is known to be a model of severe colitis that reflects human pathological features^29^. In this model, the animals were primed with either a *C. minuta* strain prepared in PBS-glycerol or the vehicle for 14 days prior TNBS injection. One group receiving the vehicle was also exposed to 5-ASA 150 mg/kg/day in food as a positive control.

**Figure 4:**
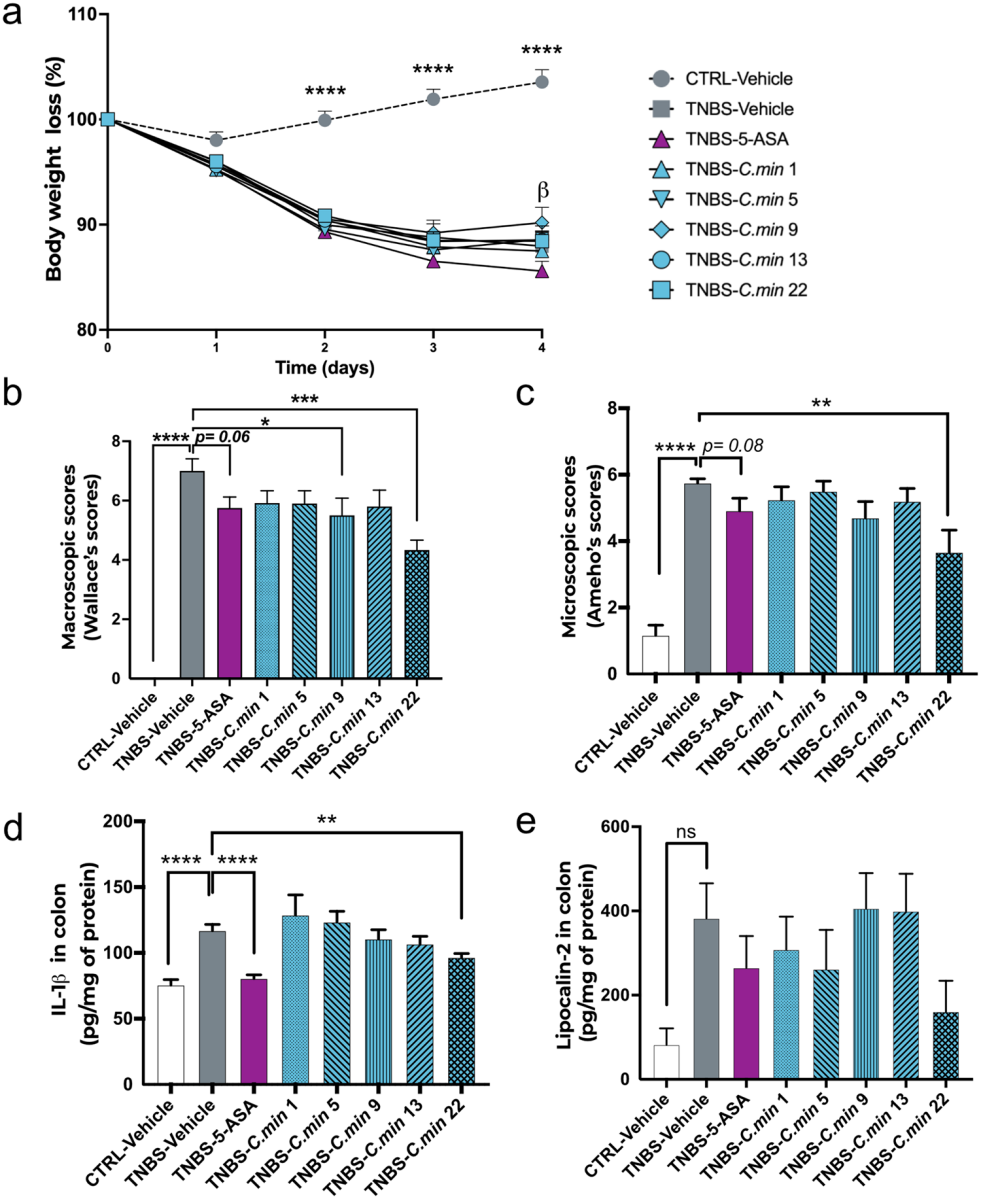
STEP 3 screening in a TNBS-induced rat colitis model. Animals were primed with either a *C. minuta* strain prepared in PBS-glycerol or the vehicle for 14 days prior TNBS injection. One group receiving the vehicle was also exposed to 5-ASA 150 mg/kg/day in food as a positive control. Body weight loss after TNBS induction (**a**; *: TNBS-Veh vs CTRL-Veh, **b**: TNBS-Veh vs TNBS-5-ASA). The effect of the different *C. minuta* strains following inflammation induced by TNBS was assessed by macroscopic score (**b**) and microscopic score (**C**) (minimum n = 6/group). Interleukin-1b (**d**) and lipocalin-2 levels (**e**) were also measured in colonic tissues. *Statistics*: repeated measures mixed model effect or Kruskal–Wallis with multiple comparisons test (**a**). Unpaired t-test or Mann Whitney test for model validation, one way ANOVA or Kruskall-Wallis with multiple comparisons for treatments effects (**b**–**e**). *p < 0.05, **p < 0.01, ***p < 0.001, ****p < 0.0001.

Following the induction of colitis, a significant decrease of the total body weight was observed in all groups receiving TNBS without significant recovery 4 days post-induction (Fig. 4a). The intensity of colonic inflammation and lesions was evaluated at the macroscopic level^14^. Out of the five tested strains, two displayed a significant improvement in colitis severity score when compared to the vehicle group (TNBS-*C. min9*: 5.50 ± 0.58 and TNBS-*C. min22*: 4.33 ± 0.33 vs TNBS-Vehicle: 7.00 ± 0.41, p = 0.02 and p = 0.0001, respectively) (Fig. 4b). *C. min *22 was also the only strain inducing a significant improvement of the inflammatory lesions at the histological level^15^ corresponding to a 36% decrease of the level of inflammation compared to TNBS-Vehicle (TNBS-*C. min22*: 3.67 ± 0.67 vs TNBS-Vehicle: 5.75 ± 1.66, p = 0.002) (Fig. 4c) and inducing a significant local anti-inflammatory effect through the reduction of colonic IL-1β protein (TNBS-*C. min22*: 96.51 ± 2.84 vs TNBS-Vehicle: 116.92 ± 4.72, p = 0.008) (Fig. 4d). Colonic levels of lipocalin 2, IL-6 and IFN-γ were not significantly induced following TNBS induction, preventing detection of treatment effects (Fig. 4e, Supplementary Fig. S2). Therefore, *C. min22* was the candidate strain displaying the best response profile in this model of colitis and was selected as the future drug candidate. The strain *C. min *22 was then deposited to the German Collection of Microorganisms and Cell Cultures (DSMZ) and registered as *C. minuta* DSM 33715.

**STEP 4: validation of the anti-inflammatory action of** ***C. minuta*** **DSM 33715 (** ***C. min*** **22) in two distinct chemically induced colitis mouse models (DSS and DNBS)**

Following STEP3 in vivo selection, we validated the anti-inflammatory effects and evaluated the potential protective effects on mucosal healing of the candidate strain DSM 33715 (*C. min* 22), in two distinct chemically induced colitis models in mice. First, DSM 33715 was assessed in a DSS-induced colitis mouse model. DSS administration induces prominent diarrhea coupled with a superficial inflammation of the gastrointestinal tract, which is usually milder than TNBS-induced colitis in the colon. In this study, mice received either *C. minuta* DSM 33715, the vehicle, or a positive control (5-ASA) for 14 days by daily oral gavage prior exposure to DSS-containing water (2.5%) for 5 days (minimum n = 6 per group). Following the DSS challenge, animals were monitored during a 6-day recovery period before euthanasia.

**Figure 5:**
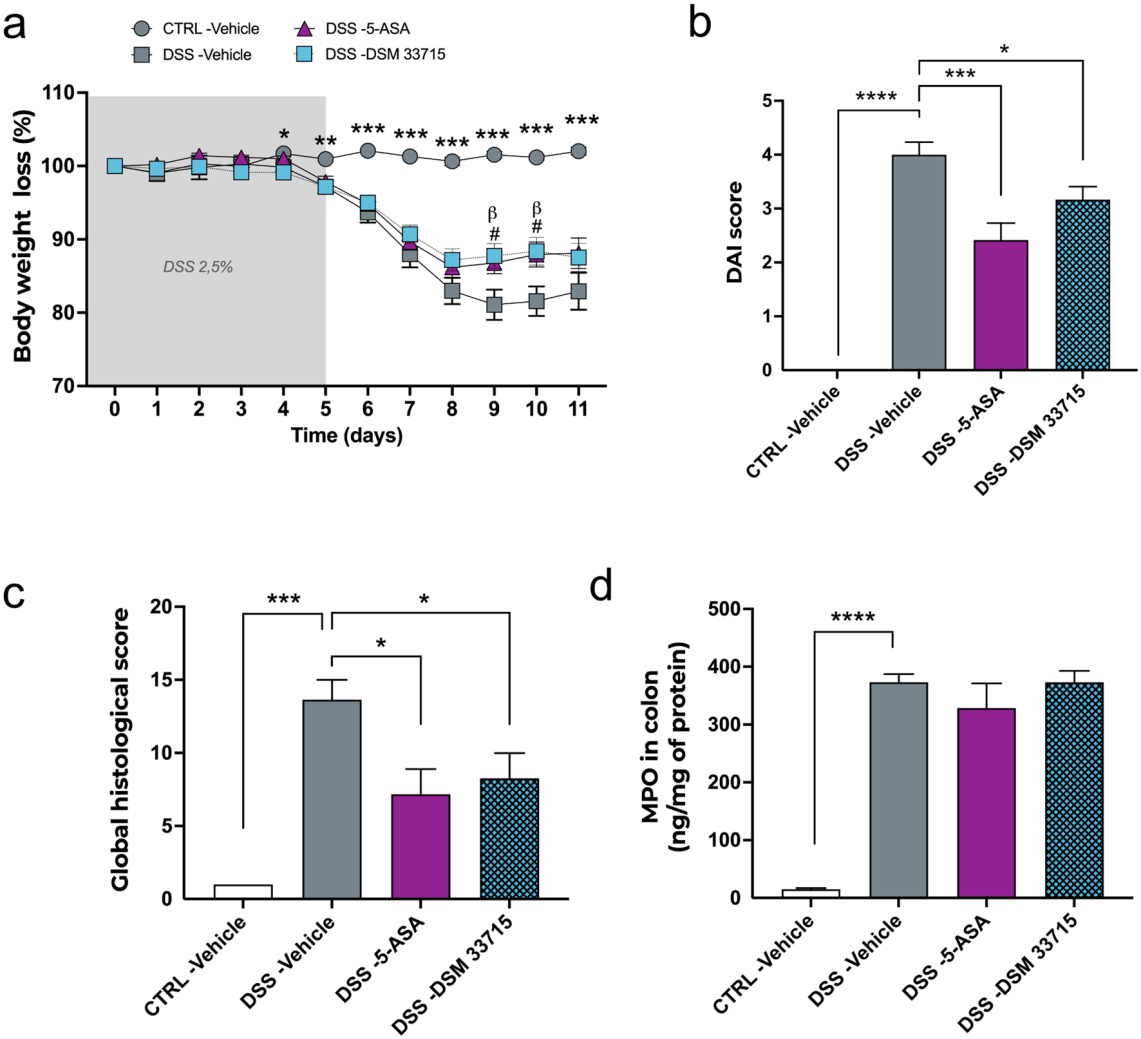
*C. minuta* DSM 33715 (*C. min* 22) protects against DSS-induced colitis in mice. Animals received either *C. minuta* DSM 33715 (*C. min* 22) (10^9^ CFU/day), the vehicle, or a positive control (5-ASA 150 mg/kg/day) for 14 days by daily oral gavage prior exposure to DSS-containing water (2,5%) for 5 days (minimum n = 6 per group). Following the DSS challenge, animals were monitored during a 6-day recovery period before euthanasia. Body weight loss was monitored for 11 days from DSS administration (**a**; *: DSS-Veh vs CTRL-Veh, (**b**): DSS-Veh vs DSS-5-ASA, #: DSS-Veh vs DSS-DSM 33715). The DAI score (**b**) and global histological score (**c**) were recorded. Colonic dosage of the MPO (**d**). *Statistics*: repeated measures mixed model effect or Kruskal–Wallis with multiple comparisons test (**a**). Unpaired t-test or Mann Whitney test for model validation, one way ANOVA or Kruskall-Wallis with multiple comparisons for treatments effects (**b**–**d**) *p < 0.05, **p < 0.01, ***p < 0.001, ****p < 0.0001.

As expected, DSS administration induced an important loss of body weight until 3 days post DSS challenge, which corresponds to the peak of inflammation. This body weight loss was significantly attenuated by 5-ASA and DSM 33715 treatments in the last days of the recovery period (Fig. 5a). Administration of 5-ASA and DSM 33715 ameliorated clinical parameters impaired by the DSS administration as indicated by improvement of the DAI score^16^ (DSS-Vehicle: 4.00 ± 0.23 vs DSS-5-ASA: 2.42 ± 0.31, p = 0.0004) and (DSS-Vehicle: 4.00 ± 0.23 vs DSS-DSM 33715: 3.17 ± 0.24, p = 0.03), respectively (Fig. 5b). The evaluation of the global histological score based on colonic sections^17^ confirmed this improvement with both treatments (DSS-Vehicle: 13.64 ± 1.36 vs DSS-5-ASA: 7.17 ± 1.72, p = 0.015) and (DSS-Vehicle: 13.64 ± 1.36 vs DSS-DSM 33715: 8.25 ± 1.72, p = 0.02), respectively (Fig. 5c). Finally, we did not observe any effects of DSM 33715 and 5-ASA on myeloperoxydase (MPO), an enzyme present in the intracellular granules of neutrophils (Fig. 5d). Together, these data support the anti-inflammatory effects of DSM 33715 observed in the rat.

**Figure 6:**
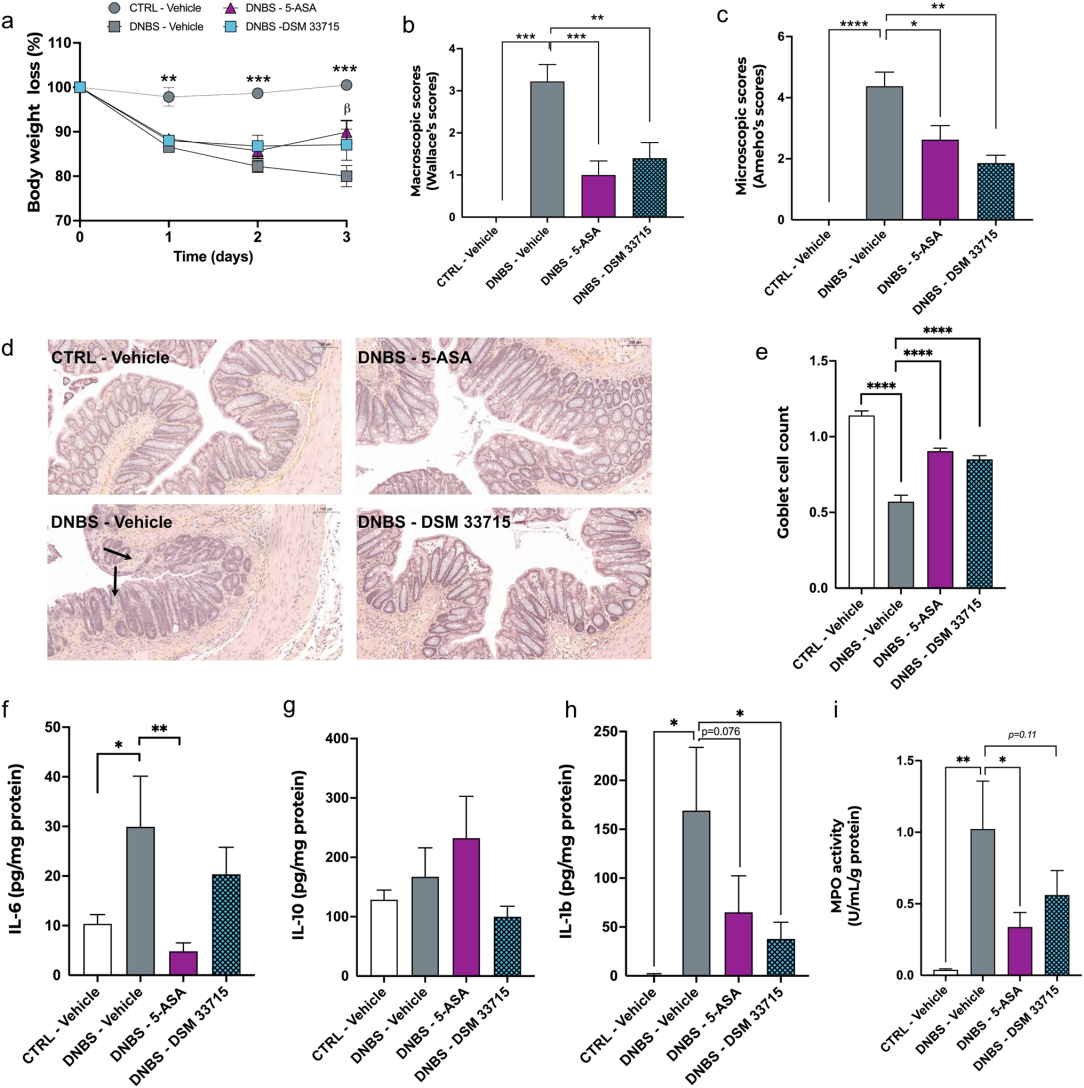
*C. minuta* DSM 33715 (*C. min* 22) protects against DNBS-induced colitis in mice. Animals received either *C. minuta* DSM 33715 (*C. min* 22) (10^9^ CFU/day), the vehicle, or 5-ASA (150 mg/kg/day) daily for 14 days by oral gavage prior intra-rectal DNBS injection (minimum n = 6 per group). The positive control 5-ASA was administered on the day of colitis induction and for the following 3 days of recovery period. Body weight loss was monitored for 3 days post DNBS injection (**a**). The macroscopic (**b**) and microscopic (**c**) scores were recorded *post mortem*. Evaluation of the colonic epithelial structure (**d**). Arrows indicate strong alterations of the epithelial surface. Goblet cell number per crypt (**e**). Colonic levels of IL-6 (**f**), IL-10 (**g**) and IL-1β (**h**) cytokines and MPO activity (**i**). *Statistics*: (**a**) Repeated measure mixed effects model followed by Dunnet’s multiple comparison test where the DNBS-vehicle group was chosen as control; (**b**,**c**) Kruskal–Wallis followed multiple comparison; (**e**–**i**) One-way ANOVA followed by multiple comparison. *p < 0.05, **p < 0.01, ***p < 0.001, ****p < 0.0001 (minimum n = 6 per group).

We further evaluated these anti-inflammatory effects in another mouse model using DNBS-induced colitis, a model close to the TNBS-rat model but in a distinct species. Mice received either DSM 33715, the vehicle, or 5-ASA daily for 14 days by oral gavage prior intra-rectal DNBS injection (minimum n = 6 per group). The positive control 5-ASA was administered on the day of colitis induction and for the following 3 days of recovery period. As expected, DNBS infusion induced a strong decrease in body weight that was attenuated by 5-ASA (DNBS-Vehicle: 80.1 ± 2.4 vs DNBS-5-ASA 89.8 ± 2.6, p = 0.01) and DSM 33715 (DNBS-Vehicle: 80.1 ± 2.4 vs DNBS-DSM 33715 87.1 ± 3.5; p = 0.1) 3 days post colitis induction (Fig. 6a). Additionally, administration of 5-ASA and DSM 33715 protected the integrity of the intestinal epithelium as indicated by the macroscopic scores^14^ (DNBS-Vehicle: 3.22 ± 0.4 vs DNBS-5-ASA: 1.0 ± 0.3 and DNBS-DSM 33715 1.4 ± 0.4; p = 0.0007 and p = 0.008, respectively) (Fig. 6b). The evaluation of microscopic scores^15^ confirmed that both 5-ASA and DSM 33715 treatments were able to significantly alleviate mucosal damage (DNBS-Vehicle: 4.38 ± 0.5 vs DNBS-5-ASA: 2.63 ± 0.5 and DNBS-DSM 33715: 1.9 ± 0.3; p = 0.03 and p = 0.001 respectively) (Fig. 6c). Observation of colonic histological sections confirmed this improvement since strong alterations of the epithelial structure were only observed in the DNBS-Vehicle group (Fig. 6d). Accordingly, the goblet cell count per crypt was also significantly improved with 5-ASA and DSM 33715 treatments (DNBS-Vehicle: 0.57 ± 0.1 vs DNBS-5-ASA: 0.9 ± 0.1 and DNBS-DSM 33715: 0.85 ± 0.13; p < 0.0001 and p < 0.0001 respectively) (Fig. 6e).

Focusing on the immune modulation, we detected a significant increase of IL-6 levels in the DNBS-Vehicle group that was only significantly reduced by 5-ASA (DNBS-Vehicle: 29.92 ± 10.2 vs DNBS-5-ASA: 4.81 ± 1.7, p = 0.0045) (Fig. 6f). No significant increases of IL-10 levels were detected in this model (Fig. 6g). However, a marked increase of IL-1β was observed, that was significantly attenuated by DSM 33715 (DNBS-Vehicle: 169.1 ± 64.7 vs DNBS-DSM 33715: 37.71 ± 17.1; p = 0.028), but not by 5-ASA (DNBS-Vehicle: 169.1 ± 64.7 vs DNBS-5-ASA: 65.07 ± 37.21; p = 0.076) (Fig. 6h). Finally, we detected a tendency for DSM 33715 to diminish colonic MPO activity (DNBS-Vehicle: 1.02 ± 0.3 vs DNBS-5-ASA: 0.33 ± 0.1 and DNBS-DSM 33715: 0.56 ± 0.2; p = 0.02 and p = 0.11 respectively) (Fig. 6i). Together, these data confirmed the anti-inflammatory capacity of the *C. minuta* candidate strain DSM 33715 to protect from damages induced by chemically-induced colitis in animal models.

To investigate potential mechanisms of action, the production of short chain fatty acids (SCFA) in the caecal content was evaluated as indicators of modulation of gut microbiota activity. As expected, the DNBS treatment induced a decrease in SCFAs production, which was largely restored by the treatments with 5-ASA and DSM 33715 (Supplementary Fig. S3). Specifically, 5-ASA and DSM 33715 significantly increased acetate, butyrate, propionate and valerate (Supplementary Fig. S3) productions. Moreover, the two branched short chain fatty acids (BSCFA) isobutyrate and isovalerate also showed an increased production upon 5-ASA and DSM 33715 treatments (Supplementary Fig. S3). Observing these positive effects in both active treatments (5-ASA and *C. minuta* DSM 33715) suggests that restoration of cecal SCFAs levels was likely the consequence of a lower inflammatory stress and overall improved gut health. Nevertheless, these data demonstrate the potential of *C. minuta* DSM 33715 to protect gut microbial metabolism from DNBS-induced damages.

### Microbiological characterisation of ***C. minuta*** DSM 33715 (*C. min* 22)

**Figure 7:**
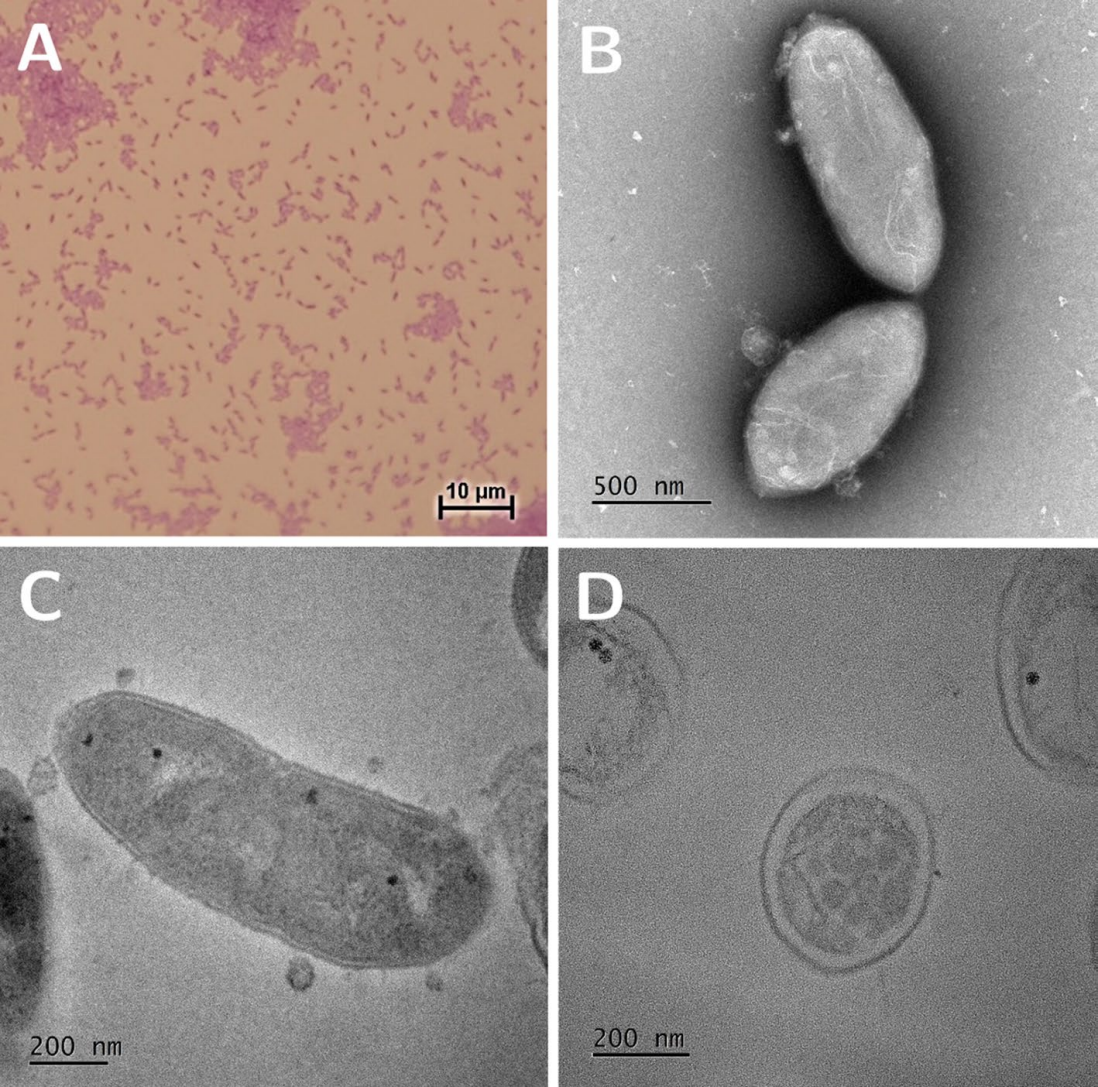
Representative pictures of *C. minuta* DSM 33715 (*C. min* 22). (**a**) Gram staining of DSM 33715; (**b**–**d**): transmission electron microscopy acquisition of DSM 33715 after simple negative coloration (**b**) or after resin embedding showing a longitudinal section (**c**) and a transversal section (**d**).

After candidate selection, the selected strain DSM 33715 (*C. min* 22) underwent a full microbiological characterisation in compliance with regulatory expectations regarding future new live biotherapeutic products. *C. minuta* DSM 33715 was strictly anaerobic, nonmotile, non-spore-forming and Gram-negative (Fig. 7a). Colonies were circular, raised, opaque, and tiny (< 1 mm). Cell morphology observed using TEM microscopy revealed short straight rods with tapered ends, as described for *C. minuta* DSM 22607^5^ and DSM 33407^18^, occurring singly or in pairs (Fig. 7b–d). Average dimensions were 1.27 ± 0.28 μm length, 0.507 ± 0.04 μm width and 29.4 nm membrane thickness (average calculated from 41 cells), which is consistent with a Gram-negative species.

In accordance with previous descriptions of *C. minuta* strains^5,18^, *C. minuta* DSM 33715 was negative for oxidase and catalase activities, and was able to metabolize glucose, xylose, arabinose, mannose, and rhamnose (Supplementary Table S1). However, this strain presented a slightly divergent biochemical profile compared to other described *C. minuta* strains since it was negative for salicin and mildly positive for mannose. Similarly to other *C. minuta* strains^5,18^, DSM 33715 was resistant up to 80 g/L Oxgall, corresponding to 80% bile (Supplementary Table S1).

Antibiotic resistance profiling was performed according to the guidelines from the Clinical and Laboratory Standards Institute (Supplementary Table S2) and revealed resistance to tetracycline and ampicillin. The main fatty acids measured in DSM 33715 were C_18:2ω9,12c_ (50.17%), C_14:0_ (28.22%), C_16:0_ (8.16%), which is a similar profile to DSM 22607 and DSM 33407 (Supplementary Table S3). However, the fatty acid profiles reported here contrast with previous analysis^5^. These discrepancies can be explained by technical differences. Indeed, in addition to different analytical methods, the source biological material was also distinct since Morotomi et al. started from solid cultures whereas here liquid cultures were used^5^.

## Discussion

This work was intended to select a *C. minuta* strain candidate as a future live biotherapeutic product using a method based on non-biased screening steps followed by in vivo validation. For this purpose, a collection of 32 *C. minuta* strains were screened based on rigorous selection criteria using in vitro and in vivo assays that allowed to identify a promising bacterial strain with high anti-inflammatory potential.

Our screening program included an evaluation of the protective action of the strains on gut epithelium upon challenge with the pro-inflammatory cytokine TNF-α. For this, we used a monolayer of human Caco-2 cells that constitute the gold standard in mimicking the human intestinal barrier (STEP 1—TEER assay). Interestingly, we observed that all tested *C. minuta* strains had a significant protective action on intestinal barrier integrity. This is consistent with other observations in similar screening assays^19^. As a consequence, it was not possible to use this assay to discriminate between strains. In addition, to favor the inclusion of genetically distinct *C. minuta* strains, we prioritized strains donor diversity to select between strains with similar scoring. We also evaluated the anti-inflammatory potential of the *C. minuta* strains using the HT-29 human intestinal cell line and human-derived PBMCs by quantifying the production of relevant cytokines. The HT-29 cells, deriving from a human colorectal adenocarcinoma, are good representatives of the intestinal membrane at both structural and functional levels^20^ and were selected here for their ability to secrete high amounts of IL-8 when challenged with TNF-α. IL-8 is a strong neutrophil chemoattractant produced by epithelial intestinal cells (EIC) that has been found overexpressed in the mucosa of all intestinal segments from the ileum to the rectum in active CD patients^21^ as well as in the serum of both CD and UC patients^22^. We therefore considered that strains with a strong ability to prevent IL-8 release would be of high therapeutic potential. Similarly, IL-10 has long been known for its anti-inflammatory action as it down-regulates the secretion of the pro-inflammatory cytokines TNF-α and IL-1β^23^. In addition, the discovery that mice lacking IL-10 and IL-10 receptor expression develop spontaneous enterocolitis established the crucial role of IL-10 in intestinal inflammation and in the etiology of IBD^24^. IL-10 being mostly produced by leukocytes, we chose to screen our collection of *C. minuta* strains on human-derived PBMC as an indicator of a systemic anti-inflammatory potential in humans.

Based on this rationale, we were able to select 5 pre-clinical candidates with high potential to protect the intestinal mucosa in IBD. We thus progressed with the selection of the clinical candidate in vivo in a chemically induced colitis model. Out of the 5 pre-selected strains, two significantly reduced the macroscopic score in the TNBS-induced colitis rat model, and only *C. min* 22 (DSM 33715) managed to successfully reduce the microscopic score (Fig. 4). On one hand, this result indicates that our screening strategy was appropriate to identify a strong clinical candidate for future drug development. On another hand, the panel of chosen in vitro assays may be complemented to consider other markers of active enterocolitis, such as reduction of TNF-α, of the interferon gamma-induced protein (IP)-10 expression or of IL-1β, which have all been reported as being massively increased in active CD biopsies^21^.

*C. min* 22 (DSM 33715), our selected candidate strain, limited clinical degradation in the DSS-induced colitis mouse model, a gold-standard for IBD modeling^25^. Interestingly, it also strongly reduced colonic IL-1β in two in vivo models suggesting that screening on the ability to reduce IL-1β secretion by macrophages in vitro might improve predictability. From a therapeutic perspective, low serum baseline concentrations of IL-1β have been associated with a higher response rate to the anti-TNF-α drug infliximab in CD patients, but not in UC^26^. This observation opens perspectives of potential use of *C. minuta* DSM 33715 (*C. min* 22) as an add-on to anti-TNF-α therapy for CD patients.

Observations made in animal models should always be interpreted with caution due to their limited ability to predict human response. Even if we have a strong rationale based on epidemiological data to target specifically CD where *Christensenellaceae* have been consistently reported as being reduced^11^, there is currently no established accurate preclinical model of CD that would discriminate from UC. For this reason, we diversified the models used and paid particular attention to use different animal species (i.e., rats and mice). We chose these chemically induced acute models of colitis primarily because they are well characterized, reproducible, simple to set up and deliver quick readouts, which are all essential criteria for a screening process. In addition, combining these models provides some interesting insights into potential mechanisms of action. Indeed, TNBS- and DNBS-induced colitis models (so called hapten-induced colitis) provoke a cell-mediated acute inflammation mostly affecting the distal colon that resembles human IBD^27^. Both TNBS and DNBS induce a similar immune response in rodents as demonstrated by Wallace et al.^28^, involving activation of the Th1- and Th17-mediated immune system^29^. To the contrary, the DSS-mouse model induces erosion of the mucosal layer in the lower part of the intestine, provoking an acute colitis through epithelial damage^27^. Although the immune response to DSS is first characterized by a strong Th1-mediated response with high TNFα secretion, it rapidly converts into a Th2-dominant response^30^. The fact that we were able to prevent gut inflammation in both models indicates that the bacteria mediate their effects through a common immune pathway that will need to be explored in the future. Nevertheless, these models mostly involve the innate immune response and therefore limit our ability to anticipate responses of the adaptive immune system^31^. Thus, follow-up studies focusing on detailed mechanisms of action will also need to consider chronic models of colitis, ideally combined with genetic models of IBD and humanized animal models to improve prediction of outcomes in patients.

From a mechanistic perspective, we observed a reduction of caecal SCFAs after DNBS-induced colitis in vehicle-treated animals, which is aligned with clinical observations where fecal SCFAs and SCFA-producing bacteria have been shown to be reduced in IBD^32^. Interestingly, both DSM 33715 (*C. min* 22) and 5-ASA prevented these colitis-induced reductions of SCFAs, which may potentially mediate the beneficial effects observed on gut inflammation in this study since SCFAs have strong immunomodulatory properties. These are mediated through stimulation of GPCR41 receptors, which induce production of protective IL22 by CD4+ T cells^33^ and GPCR43 receptors that promote Treg cells, the immune sentinels of healthy gut epithelia^34^. Accordingly, the pharmacological stimulation of SCFA receptors has also been shown to reduce susceptibility to develop colitis in a DSS mouse model through GPCR43 stimulation^35^. Even if the modulatory effect of 5-ASA on IBD patients’ gut microbiota has been documented^36^, its impact on SCFA levels has never been described to date. Considering the previously described keystone role of *C. minuta* species^18,37^, we assume that the beneficial effect of DSM 33715 (*C. min* 22) on SCFA levels is mediated through a modulation of gut microbial function leading to an increased production of SCFA. Yet, further metagenomics studies on fecal material collected during colitis studies are necessary to elucidate this point.

Finally, the selected *C. minuta* clinical candidate, strain DSM 33715 (*C. min* 22), was characterized following current regulatory guidelines appropriate for further drug development^38^. The characterization panel revealed high similarity with previously described *C. minuta* strains^5,18^ but DSM 33715 (*C. min* 22) slightly differed from the others at the biochemical level (negative for salicin and positive for mannose metabolism) but these modest differences are not sufficient to explain differences in anti-inflammatory effects. Hence, a complete comparative genome analysis will be needed to identify specific regions that may convey unique functions to this particular strain.

In conclusion, we described here a complete screening process combining in vitro and in vivo assays to conduct a rational selection of a live bacterium of the *C. minuta* species as a clinical candidate for CD therapy. Starting from a collection of 32 strains, we identified *C. minuta* DSM 33715 (*C. min* 22) as the most promising candidate. This strain will then need to be further evaluated to be granted authorization to enter clinical stage. At a broader level, such approach will be valuable to screen other live micro-organisms to be developed as future live biotherapeutic products for clinical applications.

## Methods

### Human stool collection and bacterial isolation

Human stool sample collection was authorized by the French national competent ethical authority (EudraCT number: 2020-A02134-35) under the status of Non-interventional trial involving human (RIPH3 in French classification) respecting European Directive on Clinical trials 2001/20/EC (authorization number EUDRACT (European Union Drug Regulating Authorities Clinical Trials Database) = 2020-A02134-35). Relevant data regarding volunteer health were collected following the French Reference Methodology (MR-003) in line with the European Regulation (EU-2016/679). All donors were 18–60 years old Europeans from both genders and provided written informed consent prior collection. To be included, volunteers self-declared them as healthy and free from any oral antibiotic treatment for at least 2 months before sample collection.

The strains were isolated as previously described^39,40^.

### Bacterial cell culture conditions

*Christensenella minuta* strains were cultured on pre-reduced Gifu anaerobic modified medium (GAMm, Hyserve) during 3 days at 37 °C in anaerobic atmosphere (H_2_ 5%, CO_2_ 5%, N_2_ 90%). For the screening assays, all cultures were grown until reaching an optical density of 0.3 ± 0.05. Bacterial cultures were then centrifuged and resuspended in PBS-1% glycerol before storage. All in vitro assays were performed using the strains at a multiplicity of infection (MOI) of 50 (50 bacterial cells for 1 eukaryotic cell), except for the NF-κB reporter assay that was performed using the culture supernatant. Reference strain *C. minuta* DSM 22607 was used as a control in all performed assays.

### Eukaryotic cell culture conditions

All cell lines were obtained from the European Collection of Authenticated Cell Cultures (ECACC, Sigma). HT-29 cells, Caco-2 cells and THP-1 cells were grown in the conditions described by the manufacturer. Human peripheral blood mononuclear cells (PBMCs) isolated from blood of healthy donors were obtained from Lonza Biosciences and stored in liquid nitrogen for long-term storage (> 1 month). After thawing, cells were maintained in 10%FBS-RPMI-1640 medium (Gibco) during 24 h before seeding for experimentation.

### Immunomodulatory assays in PBMCs

Assays were performed on three healthy donors. PBMCs were seeded at 1 million per well in 24-well plate. Bacteria were added at a MOI 50 or PBS-Glycerol, as a control, and co-cultures were maintained for 24 h. Interleukine-10 (IL-10) was quantified in supernatant by ELISA (human IL-10; BioLegend).

### Immunomodulatory assays in PMA-differentiated-THP1 cells

THP-1 cells were seeded at 5 × 10^5^ cells/well in 24-well plate in complete medium with 100 ng/ml PMA (Enzo Life Sciences) during 48 h, for differentiation in M0-macrophages. After differentiation, adherent M0 were washed with 2% FBS medium and co-incubated with bacteria (MOI 50) or its control (PBS-glycerol) in 2% FBS medium for 24 h. IL-10 was quantified in supernatant by ELISA (human IL-10; BioLegend).

### Immunomodulatory assays in HT-29 cells and HT-29 cells transfected with NF-κB luciferase reporter vector

HT-29 cells were used to determine effects on the regulation of inflammation induced by TNF-α stimulation by measuring IL-8 production or NF-κB activation, as described previously in Kropp et al.^12^.

### Assessment of intestinal permeability by transepithelial electrical resistance (TEER)

The Caco-2 cell line was used to determine effects on epithelial barrier function^12^. Briefly, when the optimal TEER values were reached (EVOM3, World Precision Instrument), cells were treated in the apical compartment, with bacteria at a MOI 50 or its control (culture medium), 3 h before adding 100 ng/ml of TNF-α (InvivoGen) in the basal compartment. The TEER was measured at baseline and 6 h post-treatment. Results were normalized to the basal condition.

### Animal studies

All animal studies were conducted in accredited research facilities and approved by local ethics committees in addition to the French government. TNBS and DSS studies were conducted by an accredited Contract Research Organization (Intestinal Biotech Development, Lille) in accordance with European Legislation on animal welfare. These studies were approved by the local investigational ethics review board (Nord-Pas-de-Calais CEFA N°75, Lille, France; protocol reference numbers 352012 and 19-2009R) and French government agreement (n°APAFIS#7542-20 17030609233680). The DNBS study was performed in accredited facilities of the National Research Institute for Agriculture, Food and Environment (IERP, INRAe) in accordance with European Legislation on animal welfare and were approved by COMETHEA, the local committee on animal experimentation. (ethics authorization n° APAFIS #16744 201807061805486). The following experimental protocol and associated results are reported following the ARRIVE 2.0 guideline for experimental animals.

#### TNBS-induced colitis in rats

Sprague Dawley male rats were randomly divided into 4 groups (n = 6 for the CTRL-Vehicle and n = 12 for the TNBS-induced groups). To evaluate the product effect a group TNBS-Vehicle and a positive control TNBS-5-ASA were included. The five pre-selected candidates were administered by oral gavage at 10^9^ CFU/day, starting 14 days before colitis induction until the day of euthanasia and the 5-ASA granules (150 mg/kg/day) were mixed in food. For colitis induction, rats were anesthetized and received an intrarectal injection of TNBS (80 mg/kg in 40% Ethanol). Animals were sacrificed by neck dislocation 4 days after TNBS injection and tissue samples were collected and stored at − 80 °C for analysis. The body weight variation, macroscopic and microscopic damage scores were determined as previously described^14,15^. Inflammation was assessed by measuring IL-1ß, IL-6 and IFN-γ cytokine productions (eBioscience) and the level of lipocalin-2 (Clinisciences) in colon by ELISA method.

#### DSS-induced colitis in mice

C57BL/6 male adult mice were randomly divided into 4 groups (n = 6 for the CTRL-Vehicle and n = 12 for the DSS-induced groups (DSS-Vehicle, DSS-DSM33715, DSS-5-ASA) DSS-Vehicle and a positive control DSS-5-ASA were included. *C. minuta* DSM 33715 (*C. min* 22) and 5-ASA 150 mg/kg/day were administered as described in the TNBS model. For colitis induction, the induced mice received 2.5% of DSS (45kD; TDB Consultancy AB) in their drinking water for 5-days followed by a regime of 6 days of regular water until sacrifice by neck dislocation (total of 11-days from the start of DSS induction). The tissues samples were collected and stored at − 80 °C. Body weight evolution, Disease Activity Index (DAI)^16^ and global histological score^17^ were recorded to evaluate the product effect on colonic inflammation. MPO activity was also measured by ELISA in colonic tissue (Clinisciences).

#### DNBS-induced colitis in mice

C57BL/6 male adult mice were randomly divided into 4 groups (n = 6 mice for CTRL-Vehicle mice and n = 10 mice for DNBS- induced groups. One negative control group (DNBS-Vehicle) and one positive control group (DNBS-5-ASA) were included. DSM 33715 (*C. min* 22) was administered by oral gavage at 10^9^ CFU/day, starting 14 days before colitis induction until the day of euthanasia and the 5-ASA at 100 mg/kg/day (Sigma) only from the day of the induction. For colitis induction, the mice received an intrarectal injection of Dinitrobenzene sulfonic acid (DNBS) (175 mg/kg dissolved in 30% Ethanol). Animals were sacrificed 3 days post-induction by neck dislocation and tissue samples were collected and stored at -80 °C for analysis. Inflammation was assessed by measuring the same read-outs as for the TNBS study. Additionally, the goblet cells number count was performed as previously described^41^.

### SCFA measurements

Caecal samples were extracted with water (wt g/vol) and centrifuged 15 min at 15000*g*. Supernatants were collected and deproteinized overnight at 4 °C with the addition of phosphotungstic acid (10%, Sigma). Then, samples were processed and data analyzed as described in Kropp et al.^12^.

### Microbial characterization

All phenotypic tests were performed in triplicate and reference strains *C. minuta* DSM 22607 and *Bacteroides fragilis* DSM 2151 used as controls. Biochemical characterization (API20), oxidase activity, catalase activity, Gram staining, spore forming, bile acid and pH tolerance, and antibiotic resistance were evaluated as described in Mazier et al*.*^18^. Cellular fatty acids were analyzed at DSMZ services by gas chromatography followed by Sherlock Microbial Identification (MIDI, Microbial ID).

### Transmission electron microscopy (TEM)

For single negative coloration, a negative staining of the bacterial solution was performed using Nano-Tungsten (Nano-W, Nanoprobes, LFG Distribution). For resin embedding, bacteria were defrosted slowly (at 4 °C) and fixed with 5% (v/v) glutaraldehyde in 0.2 M cacodylate buffer (pH = 7.2) mixed 50/50 (v/v) with the culture medium at 4 °C. Then samples were washed in 0.1 M cacodylate buffer, post-fixed in 1% (v/v) osmium tetroxide in cacodylate buffer 0.1 M during 2 h and washed. Samples were embedded in 1% (w/v) agarose in water, ethanol dehydrated, and embedded in a mixture of propylen oxide and epoxy resin (Epon 812; Delta Microscopy) 50/50 (v/v) for 2 h and then in 100% resin overnight at RT followed by 24–48 h at 60 °C. Samples were sliced at 70 nm thick sections and picked up on copper grids with a carbon film at the surface and stained with uranyl acetate and lead citrate. Grids were examined with a TEM (Talos F200S G2 FEG) at 200 kV, 4 K*4 K One View. TEM studies were conducted at the Bordeaux Imaging Center—Bordeaux University, a Core facility of the French network “France Bio Imaging”.

### Statistical analysis

Statistical analysis was performed using the specialist software Prism 9 (version 9.2.0, GraphPad). As a first step, distribution normality of each dataset was appreciated using a Shapiro–Wilk test. For in vitro screening assays, a one-way ANOVA test was applied followed if appropriate with a multiple comparison using the Dunnett’s test comparing all groups to a single control indicated in each figure legend. For in vivo assays, an automatic detection of outlier values was performed using the iterative Grubb’s test. Body weight data were analyzed using repeated measures mixed-effects model followed by multiple comparisons. Macroscopic, microscopic, DAI, global histological scores were analyzed using unpaired t-test, Mann–Whitney, one way ANOVA or Kruskal–Wallis test depending on conditions. All the multiple comparison were made using two-stage step-up method of Benjamini, Krieger, and Yukutieli. All values are presented as mean ± standard error of the mean.

## Supplementary Information

Supplementary Information.

## References

[1] Sairenji T, Collins KL, Evans DV (2017). An update on inflammatory bowel disease. Prim. Care Clin. Off. Pract..

[2] Frank DN (2007). Molecular-phylogenetic characterization of microbial community imbalances in human inflammatory bowel diseases. Proc. Natl. Acad. Sci..

[3] Braun T (2019). Individualized dynamics in the gut microbiota precede Crohnʼs disease flares. Am. J. Gastroenterol..

[4] van der Lelie D (2021). Rationally designed bacterial consortia to treat chronic immune-mediated colitis and restore intestinal homeostasis. Nat. Commun..

[5] Morotomi M, Nagai F, Watanabe Y (2012). Description of *Christensenella minuta* gen. nov., sp. nov., isolated from human faeces, which forms a distinct branch in the order Clostridiales, and proposal of *Christensenellaceae* fam. nov. Int. J. Syst. Evol. Microbiol..

[6] Rosa BA, Hallsworth-Pepin K, Martin J, Wollam A, Mitreva M (2017). Genome sequence of *Christensenella minuta* DSM 22607 T. Genome Announc..

[7] Pascal V (2017). A microbial signature for Crohn’s disease. Gut.

[8] Imhann F (2018). Interplay of host genetics and gut microbiota underlying the onset and clinical presentation of inflammatory bowel disease. Gut.

[9] Kennedy NA (2018). The impact of NOD2 variants on fecal microbiota in Crohn’s disease and controls without gastrointestinal disease. Inflamm. Bowel Dis..

[10] Zakrzewski M (2018). IL23R-protective coding variant promotes beneficial bacteria and diversity in the ileal microbiome in healthy individuals without inflammatory bowel disease. J. Crohns Colitis.

[11] Pittayanon R (2020). Differences in gut microbiota in patients with vs without inflammatory bowel diseases: A systematic review. Gastroenterology.

[12] Kropp C (2021). The Keystone commensal bacterium *Christensenella minuta* DSM 22607 displays anti-inflammatory properties both in vitro and in vivo. Sci. Rep..

[13] Elson CO, Sartor RB, Tennyson GS, Riddell RH (1995). Experimental models of inflammatory bowel disease. Gastroenterology.

[14] Wallace JL, Keenan CM, Gale D, Shoupe TS (1992). Exacerbation of experimental colitis by nonsteroidal anti-inflammatory drugs is not related to elevated leukotriene B4 synthesis. Gastroenterology.

[15] Ameho CK (1997). Prophylactic effect of dietary glutamine supplementation on interleukin 8 and tumour necrosis factor alpha production in trinitrobenzene sulphonic acid induced colitis. Gut.

[16] Cooper HS, Murthy SN, Shah RS, Sedergran DJ (1993). Clinicopathologic study of dextran sulfate sodium experimental murine colitis. Lab. Invest..

[17] Dieleman LA, Palmen MJHJ, Akol H, Bloemena E, Pena AS, Meuwissen SGM, Van Rees EP (1998). Chronic experimental colitis induced by dextran sulphate sodium (DSS) is characterized by Th1 and Th2 cytokines. Clin. Exp. Immunol..

[18] Mazier W (2021). A new strain of Christensenella minuta as a potential biotherapy for obesity and associated metabolic diseases. Cells.

[19] Cuffaro B (2021). Identification of new potential biotherapeutics from human gut microbiota-derived bacteria. Microorganisms.

[20] Martinez-Maqueda D, Miralles B, Recio I, Verhoeckx K (2015). The Impact of Food Bioactives on Health: In Vitro and Ex Vivo Models.

[21] Boland BS (2015). Validation of gene expression biomarker analysis for biopsy-based clinical trials in Crohn’s disease. Inflamm. Bowel Dis..

[22] Bourgonje AR (2019). A combined set of four serum inflammatory biomarkers reliably predicts endoscopic disease activity in inflammatory bowel disease. Front. Med. (Lausanne).

[23] Schreiber S, Heinig T, Thiele H-G, Raedler A (1995). Immunoregulatory role of interleukin 10 in patients with inflammatory bowel disease. Gastroenterology.

[24] Kühn R, Löhler J, Rennick D, Rajewsky K, Müller W (1993). Interleukin-10-deficient mice develop chronic enterocolitis. Cell.

[25] Eichele DD, Kharbanda KK (2017). Dextran sodium sulfate colitis murine model: An indispensable tool for advancing our understanding of inflammatory bowel diseases pathogenesis. World J. Gastroenterol..

[26] Lacruz-Guzmán D (2013). Influence of polymorphisms and TNF and IL1β serum concentration on the infliximab response in Crohn’s disease and ulcerative colitis. Eur. J. Clin. Pharmacol..

[27] Randhawa PK, Singh K, Singh N, Jaggi AS (2014). A review on chemical-induced inflammatory bowel disease models in rodents. Korean J. Physiol. Pharmacol..

[28] Wallace JL, Le T, Carter L, Appleyard CB, Beck PL (1995). Hapten-induced chronic colitis in the rat: Alternatives to trinitrobenzene sulfonic acid. J. Pharmacol. Toxicol. Methods.

[29] Brenna Ø (2013). Relevance of TNBS-colitis in rats: A methodological study with endoscopic, histologic and transcriptomic characterization and correlation to IBD. PLoS One.

[30] Alex P (2009). Distinct cytokine patterns identified from multiplex profiles of murine DSS and TNBS-induced colitis. Inflamm. Bowel Dis..

[31] Koboziev I, Karlsson F, Zhang S, Grisham MB (2011). Pharmacological intervention studies using mouse models of the inflammatory bowel diseases: Translating preclinical data into new drug therapies. Inflamm. Bowel Dis..

[32] Parada Venegas D (2019). Short chain fatty acids (SCFAs)-mediated gut epithelial and immune regulation and its relevance for inflammatory bowel diseases. Front. Immunol..

[33] Yang W (2020). Intestinal microbiota-derived short-chain fatty acids regulation of immune cell IL-22 production and gut immunity. Nat. Commun..

[34] Smith PM (2013). The microbial metabolites, short-chain fatty acids, regulate colonic Treg cell homeostasis. Science.

[35] Agus A (2016). Western diet induces a shift in microbiota composition enhancing susceptibility to adherent-invasive *E. coli* infection and intestinal inflammation. Sci. Rep..

[36] Xu J (2018). 5-Aminosalicylic acid alters the gut bacterial microbiota in patients with ulcerative colitis. Front. Microbiol..

[37] Déjean G (2021). Identifying a novel bile salt hydrolase from the keystone gut bacterium *Christensenella minuta*. Microorganisms.

[38] Paquet J-C (2021). Entering first-in-human clinical study with a single-strain live biotherapeutic product: Input and feedback gained from the EMA and the FDA. Front. Med..

[39] Bellais S (2022). Species-targeted sorting and cultivation of commensal bacteria from the gut microbiome using flow cytometry under anaerobic conditions. Microbiome.

[40] Lagier J-C (2016). Culture of previously uncultured members of the human gut microbiota by culturomics. Nat. Microbiol..

[41] Wrzosek L (2013). *Bacteroides thetaiotaomicron* and *Faecalibacterium prausnitzii* influence the production of mucus glycans and the development of goblet cells in the colonic epithelium of a gnotobiotic model rodent. BMC Biol..

