# Supplementary material for: Selection of a novel strain of Christensenella minuta as a future biotherapy for Crohn’s disease
Source: Sci Rep. 2022 Apr 11;12:6017. doi: 10.1038/s41598-022-10015-3 (PMC9001714; doi:10.1038/s41598-022-10015-3)
Supplement: Supplementary file 1 — Supplementary Information. [file 41598_2022_10015_MOESM1_ESM.docx]

Selection of a novel strain of *Christensenella minuta* as a future biotherapy for Crohn’s Disease

Karima Relizani ^1,#^, Katy Le Corf ^1,#^, Camille Kropp ^1,2^, Rebeca Martin-Rosique ^2^, Déborah Kissi ^1^, Guillaume Déjean ^1^, Lisa Bruno^1^, Ccori Martinez ^1^, Georges Rawadi ^1^, Frédéric Elustondo ^1^, Wilfrid Mazier ^1^, Sandrine P. Claus ^1,*^

^1^Ysopia bioscience, 17 place de la Bourse, 33076 Bordeaux, France

^2^Micalis Institute, AgroParisTech, INRAE, Université Paris-Saclay, 78350 Jouy-en-Josas, France
^*^corresponding author: Sandrine.claus@ysopia.bio
^#^these authors contributed equally to this work

**SUPPLEMENTARY INFORMATION**

a

b

**Figure S1**: Step 2 – Validation of the anti-inflammatory action and gut barrier protection of the five pre-selected candidate strains. a. IL-8 production by HT-29 cells following TNFa stimulation. b. Normalized transepithelial electrical resistance (TEER) following TNFa stimulation. Statistics: one way ANOVA followed by Dunnett’s multiple comparisons test using the Ctrl + TNF group as control (****p<0.0001; ***p= 0.0004 for *C.min* 1 and DSM 22607; p=0.0006 for *C.min* 22; **p=0.0026 for *C.min* 9 and p=0.0066 for *C.min* 5).


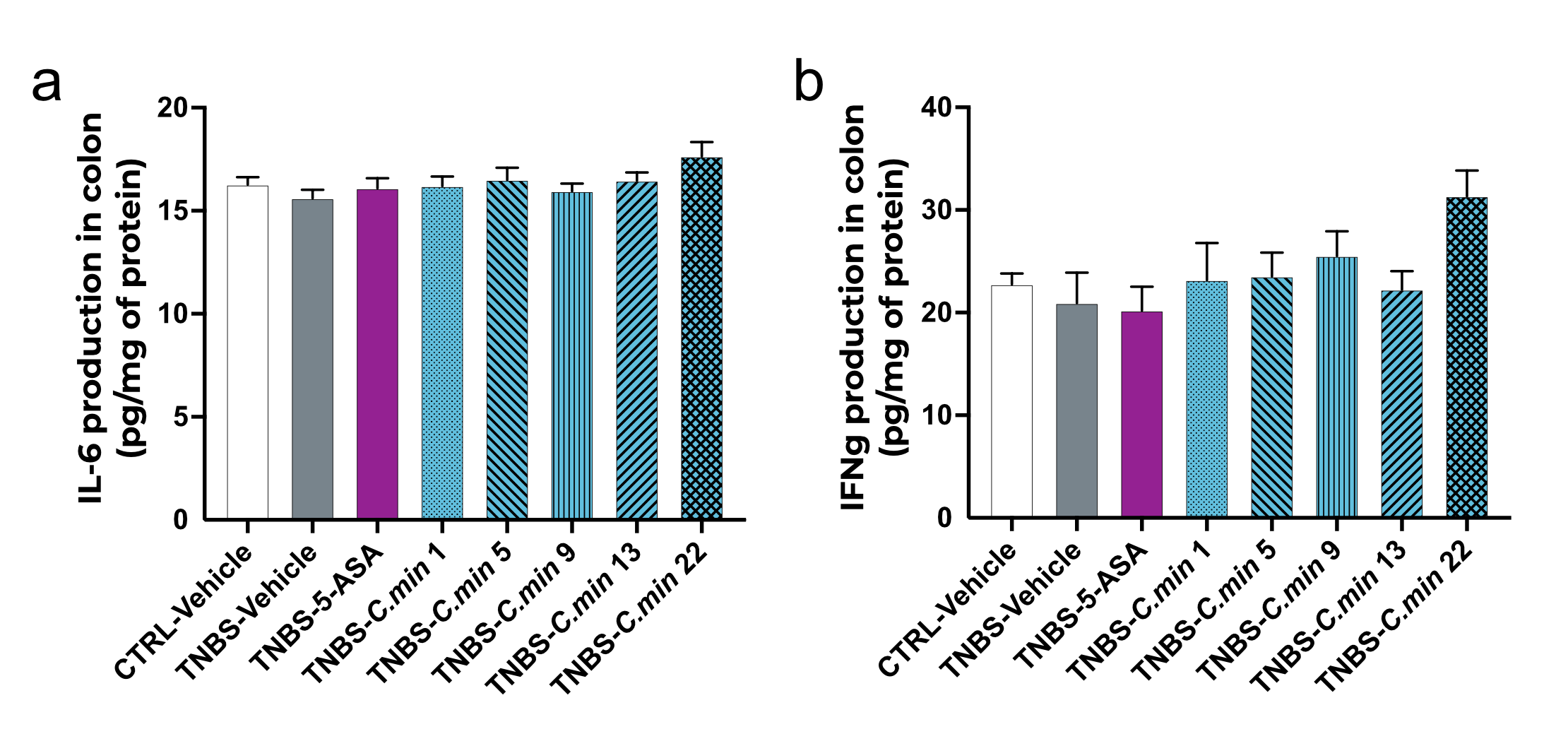


**Figure S2**: Colonic levels of Interleukin-6 (a) and Interferon-γ (b) measured in TNBS-induced colitis rats (n=12 for all groups except CTRL-Vehicle). *Statistics*: One-way ANOVA.


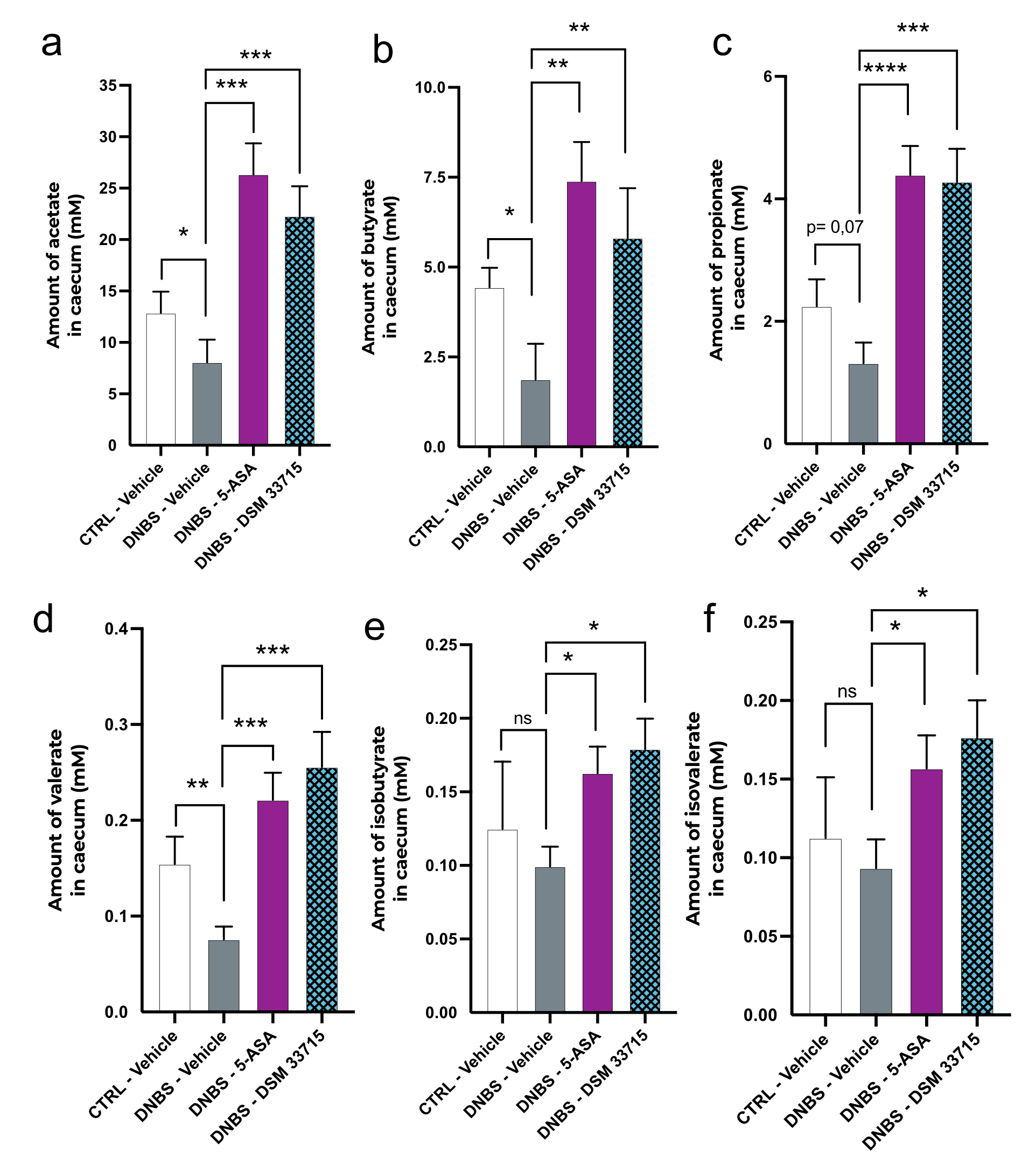


**Figure S3**: *C. minuta* DSM 33715 (C. min 22) restored caecal levels of SCFAs following DNBS-induced colitis in mice. Acetate (a), butyrate (b), propionate (c), valerate (d), isobutyrate (e) and isovalerate (f) productions in caecum. Statistics: One-way ANOVA followed by Kruskal-Wallis multiple comparison test. *p< 0.05, **p< 0.01, ***p< 0.001, ****p<0.0001 (minimum n=6 per group).

**Table S1***.* Physiological and biochemical properties of *C. minuta* DSM 33715 compared to DSM 33407 and DSM 22607.

|  | Strain characteristics | | |
| --- | --- | --- | --- |
| Morphology | | Short rods with tapered ends; single, pairs or rosettes | |
| Growth condition | | Anaerobic | |
| Gram stain | | Negative | |
| Motility | | None | |
| Spore formation | | None | |
| Catalase | | Negative | |
| Oxidase | | Negative | |
| pH range | | 6.0 to 9.0 | |
| Bile resistance | | Up to 80% | |
|  | |  |  |
|  | **API 20A Gallery results** | | |
|  | **DSM 33715** | **DSM 33407** | **DSM 22607** |
| Indole (IND) | - | - | - |
| Urea (URE) | - | - | - |
| Glucose (GLU) | + | + | + |
| Mannitol (MAN) | - | - | - |
| Lactose (LAC) | - | - | - |
| Saccharose (SAC) | - | - | - |
| Maltose (MAL) | - | - | - |
| Salicin (SAL) | - | -/+ | -/+ |
| Xylose (XYL) | + | + | + |
| Arabinose (ARA) | + | + | + |
| Gelatin (GEL) | - | - | - |
| Esculin (ESC) | - | - | - |
| Glycerol (GLY) | - | - | - |
| Cellobiose (CEL) | - | - | - |
| Mannose (MNE) | -/+ | - | - |
| Melezitose (MLZ) | - | - | - |
| Raffinose (RAF) | - | - | - |
| Sorbitol (SOR) | - | - | - |
| Rhamnose (RHA) | + | + | -/+ |
| Trehalose (TRE) | - | - | - |

**Table S2***.* Antibiotic resistance profile of *C. minuta* DSM 33715 in comparison to DSM 33407 (Mazier et al, 2021) and DSM 22607. Minimum inhibitory concentration (MIC, in mg/mL); + = sensitive; - = resistant; ± = intermediate

|  | DSM 33715 | | DSM 33407 | | DSM 22607 | |
| --- | --- | --- | --- | --- | --- | --- |
| Antibiotic Name | MIC | Sensitivity | MIC | Sensitivity | MIC | Sensitivity |
| Ampicillin | 4 | - | 4 | - | 4 | - |
| Ceftriaxone | 16 | + | 32 | ± | 32 | ± |
| Chloramphenicol | 16 | ± | 16 | ± | 16 | ± |
| Clindamycin | 0.06 | + | 0.03 | + | 0.06 | + |
| Meropenem | 0.25 | + | 0.25 | + | 0.25 | + |
| Metronidazole | 0.5 | + | 0.25 | + | 0.25 | + |
| Moxifloxacin | <0.12 | + | 1 | + | <0.12 | + |
| Piperacillin/Tazobactam | 8/4 | + | 8/4 | + | 8/4 | + |
| Tetracycline | >32 | - | >32 | - | >32 | - |

**Table S3.** Cellular fatty acids composition of *C. minuta* DSM 33715 compared to other the profiles of other *C. minuta*: DSM 33715 ^14^ and DSM 22607 ^5^. *tr*: traces below limit of quantification

| **Fatty Acids** | ***Christensenella minuta* strain** | | |
| --- | --- | --- | --- |
|  | **DSM 33715** | **DSM 33407** | **DSM 22607** |
| **Saturated straight-chain** | | | |
| **C_9:0_** | tr | tr | tr |
| **C_10:0_** | 0.58 | *0.96* | 0.59 |
| **C_12:0_** | 0.43 | *2.19* | 0.53 |
| **C_14:0_** | 28.22 | *35.55* | 31.18 |
| **C_16:0_** | 8.16 | *7.35* | 5.82 |
| **C_18:0_** | 2.51 | *3.10* | 2.20 |
| **Unsaturated straight chain** | | | |
| **C_18:1_*_w9c_*** | 50.17 | *36.09* | 50.50 |
| **C_18:2_*_w9,12c_*** | 1.83 | *1.16* | 1.87 |
| **Saturated branched-chain** | | | |
| **iso-C_11:0_** | 0.58 | *1.57* | 0.32 |
| **iso-C_15:0_** | 2.96 | *6.89* | 2.25 |
| **anteiso-C_15:0_** | 0.37 | *0.80* | 0.35 |
| **iso-C_17:0_** | 0.46 | *0.36* | 0.35 |
| **Dimethylacetal (DMA)** |  |  |  |
| **iso-C_15:0_DMA** | 0.20 | *0.70* | 0.20 |
